# Measurement and simulation of steered acoustic fields generated by a multielement array for therapeutic ultrasound

**DOI:** 10.1121/10.0003210

**Published:** 2021-01-11

**Authors:** Eleanor Martin, Morgan Roberts, Bradley Treeby

**Affiliations:** 1 Wellcome/EPSRC Centre for Interventional & Surgical Sciences (WEISS), University College London, London, United Kingdom; 2 Department of Medical Physics and Biomedical Engineering, University College London, London, United Kingdom

## Abstract

Modelling of fields generated by therapeutic ultrasound arrays can be prone to errors arising from differences from nominal transducer parameters, and variations in relative outputs of array elements when driven under different conditions, especially when simulating steered fields. Here, the effect of element size, element positions, relative source pressure variations, and electrical crosstalk on the accuracy of modelling pressure fields generated by a 555 kHz 32-element ultrasonic array were investigated. For this transducer, errors in pressure amplitude and focal position were respectively reduced from 20% to 4% and 3.3 mm to 1.5 mm using crosstalk prediction, and experimentally determined positions.

## 1 Introduction

Ultrasonic transducers used for therapeutic applications employ multiple elements to enable focal steering, and for transcranial applications, in particular, to facilitate aberration correction and focussing in the brain.^1,2^ In order to perform accurate targeting and prediction of the resulting dose to the target tissues, these complex sources and the generated fields must be modelled accurately. The most flexible option for defining the source transducer in a model is to characterise the elements under one particular drive condition, and then to apply amplitude and phase offsets in simulation to generate different steered, focused, and aberration corrected fields.^3^ A comprehensive measurement based characterisation can provide an accurate source definition, compared to assuming ideal behaviour and/or a source matching the manufacturer’s nominal parameters.^4–7^ However, the challenge still remains that the relative outputs of array elements under one set of driving conditions are not necessarily consistent with their outputs under other driving conditions. For example, differences have been observed between the output of array elements driven individually compared to when they were driven simultaneously^8^ and the output power of a 256-element clinical array transducer was approximately 5% higher when the beam was steered compared to when geometrically focused.^9^

These variations in output may arise for a variety of reasons, including the behaviour of power amplifiers, electrical crosstalk, or mechanical coupling, and vary depending on the system construction and components.^8,10–12^ Obtaining a full measurement based source definition for each possible field is not feasible, so approaches are needed for reducing uncertainty in modelling these transducers under the variety of driving conditions that may be used.

In this study, we compare simulations and measurements of steered fields generated by a 555 kHz 32-element ultrasonic array transducer, driven with a Verasonics multichannel drive platform. An investigation of the contribution to differences between measured and simulated fields is made by quantifying the effects of incorporating optimised/measured element size and positions, inter-element variations in acoustic output, and electrical crosstalk between channels with the aim of establishing the best approach for accurate simulation of fields generated by a real array source when the exact source conditions have not been characterised for every case.

## 2 Transducer properties

The transducer was assembled from 32 individual 3 mm diameter plane circular piezo-ceramic elements (XDR107, Sonic Concepts, Bothell, WA). The elements had a fundamental frequency of 555 kHz. The elements were arranged in a pseudorandom configuration in a three-dimensional (3D) printed spherical cap holder, with radius of curvature 80 mm and aperture diameter 70 mm. This holder was printed in VeroClear and mounted to a housing printed from polylactic acid. The elements were driven by a Verasonics multichannel drive platform (Vantage 256 with HIFU transmit configuration, Verasonics Inc., Kirkland, WA). Each drive channel was connected to an element via a standard ultrasound connector (DL5, ITT Cannon, White Plains, NY), in-house impedance matching network, and a multichannel twisted pair cable 10 m in length (to enable use in an Magnetic resonance imaging (MRI) scanner), which was connected via a custom breakout printed circuit board to a 0.5 m micro-coax cable running into the element.

## 3 Modelling elements as circular pistons

The elements were modelled as a set of identical circular piston radiators, the diameter of which was obtained by minimising the difference between the simulated field and the measured fields. For these measurements, 16 elements were mounted in a 3D printed holder in a planar square grid pattern, with a 12 mm centre-to-centre spacing. Drive waveforms were generated in the Verasonics control software using the “parametric” waveform type with the TW (transmit waveform) object to obtain a sinusoidal signal, with a frequency of 555 kHz and 22 cycles, which was sufficient for the field to reach steady state at all measurement positions. The amplitude of the sinusoidal drive voltage was 15 V, and the apodisation factor was 1 with zero phase (i.e., no amplitude or phase adjustments were applied). The elements were mounted in an automated scanning tank filled with degassed, de-ionized water. The acoustic fields were measured with a calibrated 0.2 mm polyvinylidene fluoride needle hydrophone (Precision Acoustics, Dorchester, UK) positioned with a three-axis (*X*, *Y*, and *Z)* computer-controlled translation stage. Waveforms were acquired, digitised, and stored via a digital phosphor oscilloscope (DPO5034B, Tektronix U.K. Ltd., Berkshire, UK) controlled via the scanning tank software, with a sample rate of 50 MS/s and 32 averages.

Measurements were made over a plane approximately perpendicular to the beam axes of the elements at an axial distance of 28 mm and with a width of 106 mm, which was sufficient to contain the lateral extent of the fields of all elements, and a spatial step size of 1 mm. The hydrophone signal was acquired in a time window occurring after signals from all parts of the elements had arrived, but before reflections reached the hydrophone (50–56*μs*). Signals were cropped to a whole number of cycles, then bandpass filtered (–6 dB passband: 200kHz–1MHz) before the frequency response of the hydrophone was deconvolved. The amplitudes and phases of the pressure waveforms at the driving frequency were then obtained from the fast Fourier transform (FFT).

The optimal element size was obtained by iteratively updating the array position and element size (via a parameter sweep) to minimise the *L2* error between the simulated and measured acoustic field over a plane 12 mm from the source (see Sec.7 for details of the simulation model). The optimised element size was 3.2 mm. This small difference may arise due to the element construction and manufacturing tolerances, as well as deviations from ideal source behaviour.

## 4 Transducer characterisation

Acoustic holography was used to characterise the outputs and positions of the array elements in the pseudo-random bowl configuration under three different conditions. The transducer was mounted in a fixed position in an automated scanning tank. Measurements of the acoustic field were made with elements driven with a 35 cycle burst, drive voltage amplitude of 8 V, and apodisation 1 for all elements, with the following phase settings: (1) phase 0 for all elements; (2) phases applied such that waveforms emitted from each element driven individually would be in phase at the geometric focus of the array; and (3) with equalised phases as for (2) plus an extra phase offset which steered the focus 20 mm off axis in both the *x* and *y* directions. To calculate the equalised phases, the hydrophone was aligned to the position of spatial peak pressure in case (1). Each element was then driven individually in sequence with a 15 V drive voltage amplitude, 30 cycles, and a 10 ms burst period, and signals were acquired with 400 averages. The phase of each waveform was extracted from the FFT of the waveforms, and offsets were calculated in reference to the minimum phase value across all waveforms. Phase offsets required for steering were calculated from the relative distances between the nominal position of each element and the desired focal position.

For each driving condition, acoustic signals were measured on a 141 mm by 141 mm plane perpendicular to the beam axis, with a spatial step size of 1 mm. For the first two cases, the measurement plane was at a distance of 56 mm from the centre of the array surface, and in the third case it was at a distance of 50 mm. Waveforms were acquired and processed to obtain the amplitude and phase of the acoustic pressure as described in Sec.3. The complex pressure plane was spatially upsampled by a factor of 2 (dx =0.5 mm) then the 3D pressure volume was projected using the angular spectrum method.

To obtain the element positions, the pressure was extracted from within a spherical shell extending a radial distance of 6 mm either side of the surface containing the nominal element positions. This volume contained the field close to each element which was ellipsoidal in shape and symmetrical about the centre. The MATLAB function REGIONPROPS3 was then used with the binarised pressure magnitude to obtain the centroid of each field. A threshold of ~0.3 was suitable for identifying all 32 elements. To further refine the centroid positions, the pressure magnitude within the bounding box of each of the fields was then individually thresholded at 80% of the maximum pressure in that region, and the centroid was extracted again. A spherical surface was then fitted to the resulting positions to obtain the radius of curvature of the array and position of the origin. This revealed that the actual element positions differed from the expected positions: the radius of curvature of the fitted surface was 83.6 mm as compared to the expected value of 80 mm. This difference likely arose from 3D printing tolerances and the assembly process, among other causes.

To obtain the magnitude and phase of the pressure at the surface of each element (assuming for these purposes that their surfaces coincided with the centroids of the ellipsoidal fields), the Rayleigh integral was used to calculate the pressure on the fitted surface. Again, the centroids of each field were obtained, this time two-dimensionally (2D) using the regionprops function. The mean magnitude and phase of the pressure of each element were then extracted over a 1.6 mm diameter disk placed at the centroid of each field. It was observed that there were significant differences in relative output of the elements between the three driving conditions. There were differences of up to 59% in the source pressure magnitude and the variance of source pressure magnitudes increased by a factor of 1.9 when the array was steered compared to when no phase corrections were applied.

## 5 Electrical crosstalk prediction

It has been previously observed that the output of array elements may differ when driven in parallel to other elements compared to when driven individually.^8^ For this transducer, this behaviour was thought to be dominated by electrical coupling between the long parallel cables making up each channel. To quantify the effect of this on simulated steered fields for this transducer, the acoustic outputs of the elements including electrical crosstalk were predicted from the electrical inputs using a 32 × 32 transfer matrix,

(1) Mmn=θcme(−Re(f)dnm)e(−iIm(f)dmn).

Here, *c_m_* are the relative complex outputs of each element when driven individually, which account for differences in output mainly due to differences in their electrical impedance. These were obtained from the single point waveform acquisitions used for phase equalisation in Sec. 4. *d_nm_* are the average distances between each channel and every other channel in the multichannel twisted pair cable. These were measured from a cross section of a spare length of the same cable at several locations. *θ* is a constant which scales the input apodisation factor and drive voltage to the output pressure. This expression assumes the electrical crosstalk can be modelled by an amplitude term (given by the real part of *f*) that decays exponentially with the distance between the wires (consistent with capacitive coupling), and a phase term (given by the imaginary part of *f*) that depends linearly on the distance between the wires. Measurement of the actual crosstalk voltages on a sub-set of the elements for different driving conditions (measurements not shown) confirmed this to be a reasonable approximation.

The MATLAB fminsearch function was used to obtain values for *θ*,*f_real_*, and *f_imag_*, by minimising the sum of squared differences between the measured acoustic output, *A_meas_*, and the transfer matrix multiplied by the electrical inputs *E*,

(2) $$\epsilon =\sum {\left(A_{meas}-ME\right)}^{2}.$$

The errors were summed across the three elements for each of the three driving conditions measured in Sec. 4, and the differences were calculated separately for amplitude and phase of the inputs and outputs. The following values were obtained: *θ* = – 288.1 kPa, *f* = 1544 – 906.8*i*.

## 6 Validation measurements

To quantify the accuracy of simulations of steered fields where the transducer is driven under conditions not explicitly characterised, a set of validation measurements was made of fields with the focus steered to a further eight positions in addition to (2) and (3) described in Sec. 4: axially to 70 and 100 mm, laterally in the *x* direction by 5, 10, 20, and 40 mm and in the *y* direction by 10 mm, and laterally in both *x* and *y* by 20 and 40 mm. The transducer was driven as described in Sec. 4 with phases calculated from the relative distances between the nominal positions of the elements and the desired focal position. Sets of lateral and axial line scans passing through the focus were obtained for each position with a step size of 0.5 mm and a length of 20 mm. Waveforms were acquired and processed as described above.

## 7 Simulation of steered fields

Simulations were performed using the acousticFieldPropagatorC function in k-Wave^13,14^ on a 0.2 m ×0.2 m ×0.2 m domain with a spatial step size of 0.5 mm. The array was defined using the kWaveArrayclass as a set of off-grid disk elements centred at the element positions, with surface normals coincident with the geometric centre of the array.^15^ For each of the ten focal positions, 12 simulations were run with permutations of the following: element size taken from the manufacturer’s specification (3 mm) and the optimised diameter (3.2 mm); element positions taken from the array design and the experimental characterisation; with the same source pressure applied to each element, with source pressures obtained by holography [case (2)], and with crosstalk prediction calculated using *ME*, where the input apodisation factor (the amplitude of *E*) was 1 for all elements. For the three different source pressure conditions, element phases required for steering were calculated as described in Sec. 4, and these were set as the phase of *E* where crosstalk was included. For each of the 12 sets of simulations, an additional scaling factor was applied to normalise the simulated focal pressure of the unsteered field to the focal pressure obtained from angular spectrum projection of the measured unsteered field [case(2)]. The same scaling factor was then applied for the other nine focal positions.

## 8 Evaluation of errors

For each simulation, the difference between the measured and simulated focal pressure amplitude, the euclidean distance between the focal positions, and the relative *l*_2_ error norm over the measured scan lines were calculated. The position of the focus was taken as the position of the spatial peak pressure amplitude in the region surrounding the intended focal position.

## 9 Results and discussion

**Fig. 1:**
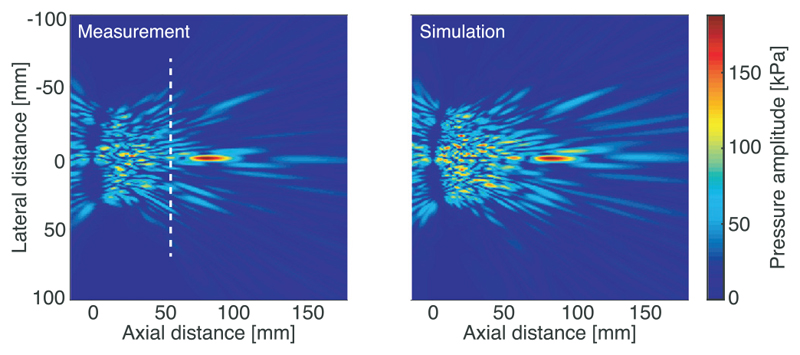
Axial-lateral plane through the measured and simulated geometrically focussed fields. Here, the measured field is the angular spectrum projection of the measured lateral plane (the dashed white line shows the position of the measured plane), and simulation is the simulated field using optimised element size, measured element positions, and crosstalk prediction.

**Fig. 2:**
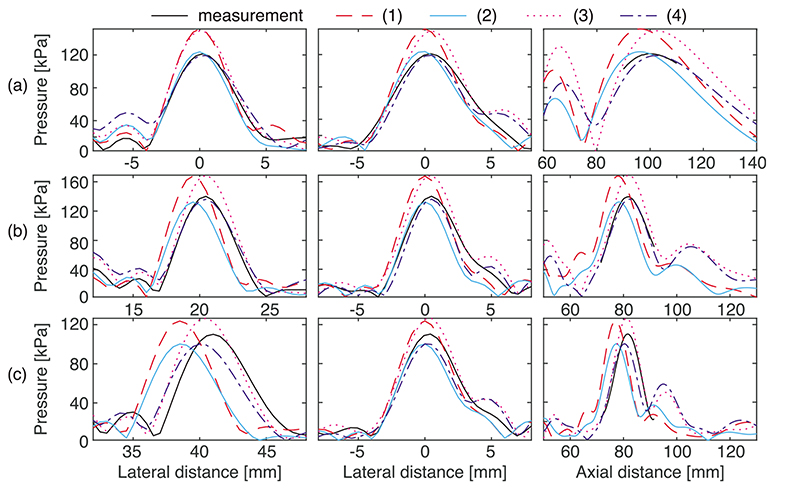
Lateral (left, middle) and axial (right) line scans passing through the focus of the measured (black line) and simulated fields with four different parameter combinations steered to (a) 0, 0, 100 mm, (b) 20, 0, 80 mm, and (c) 40, 0, 80 mm. (1) Nominal element size and positions, uniform source pressure; (2) optimised size, nominal positions, crosstalk prediction; (3) nominal size, measured positions, uniform source pressure; (4) optimised size, measured positions, crosstalk prediction.

Table 1 shows the error metrics averaged over the ten steered fields for each of the 12 simulations for which the permutations of element size, position, and source pressure are given. Figure 1 shows a lateral axial plane through the focus of the unsteered field projected from the measured data and simulated using the measured element positions, optimised element size, and crosstalk prediction. There is good qualitative agreement between the fields with similar sidelobes and near field features. Figure 2 shows comparisons between measurements and simulations performed with four different parameter sets for three steered fields that include those with the largest differences in focal position [Fig. 2(a)], amplitude [Fig. 2(c)], and smallest *l*_2_ error [Fig. 2(b)].

It can be seen that predicting the effects of crosstalk on the source pressures of the elements was important and effective, reducing the average difference between measured and simulated focal pressure from approximately 20% to approximately 4%. Without crosstalk prediction, the pressure was consistently overestimated and individual differences varied between 15% and 30%, which were reduced to within 6% with crosstalk prediction for all fields except for lateral steering of 40 mm [Fig. 2(c)] but there was no consistent pattern of increasing error with steering distance. From our previous work, the expected uncertainty on measurements of acoustic pressure amplitude is in general 10%, but for linear quasi continuous wave (QCW) fields it is closer to 6%.^16^ The difference between the measured and simulated focal pressure amplitudes were within this uncertainty when crosstalk prediction was employed, verifying this approach for accurate prediction of the pressure amplitudes.

Using the experimentally characterised element positions was important for reducing the error in focal position, reducing it from between 3.1 and 3.3 mm to between 1 and 1.5 mm. When the nominal element positions were used for the simulation, crosstalk prediction made a small improvement to the average error in focal position (3.3 to 3.1mm). When the measured positions were used, crosstalk prediction actually increased the average error by a few tenths of a mm compared to the other source pressure methods. With the measured positions, the largest difference in focal position was 2.8mm for the field steered axially to *x*, *y*, *z* = 0, 0, 100mm [Fig. 2(a)]. The smallest error was 0.5 mm for the field steered to 20, 20 mm. These focal position errors are larger than those observed in some of our previous work with single element focussing transducers, where the focal position was predicted with a 0.1mm error.^17^ In that case, the simulation was based upon characterisation of a single element, with a simple relationship between drive voltage amplitude and source pressure which was not influenced by other drive circuits. With the transducer used in the present study, there are many more sources of uncertainty, for example, the representation of all elements by the same ideal radiator, and the influence of crosstalk between channels. It is known that foci can be produced effectively with low phase resolution,^18^ which may explain why foci were still obtained close to their intended positions for this transducer despite the significant influence of crosstalk which causes deviations from the set values in phase as well as amplitude for the steered fields.

The *l*_2_ error is influenced by both the amplitude and spatial distribution of the pressure, and was reduced both by using the measured positions and by crosstalk prediction. The error was halved when both of these were used for the simulation as compared to when the nominal positions and uniform source pressure were used. Use of the optimised element size had little effect and actually increased the *l*_2_ error from 0.35 to 0.37 when crosstalk prediction and measured positions were used. The optimised element diameter was obtained from the average of a number of measurements, which showed some variation between elements. This, coupled with the similarity of the optimised and nominal element diameters, may explain the small influence of element size on the error metrics in this case. The sources were modelled as a uniform injection of mass in a free space (equivalent to uniform source velocity), which for these transducers produces a gaussian like source pressure distribution.^6^ This agreed well with the source surface pressure distributions obtained experimentally for Sec.3. For transducers with larger ka values, there are likely to be other features arising from surface waves, which if neglected could lead to errors in the field distribution and focal position.^6,11^

Outside of the focal region, the background is more featured than the field of a single element focussing transducer, or fields generated by more fully populated arrays. These features were observed to vary depending on the element size and positions, but remain low amplitude compared to the focus and would decrease further with the addition of more elements to the array.^19^ The main aim of this study was to predict the position and amplitude of the focal region accurately under different driving conditions so these background variations have not been studied here.

## 10 Conclusion

For this particular transducer, including the effect of electrical crosstalk on the acoustic output was necessary for accurate simulation of steered fields where the source pressures of each element had been characterised under a different set of drive conditions. Electrical crosstalk prediction reduced the error in the focal pressure amplitude from around 20% to approximately 4%. To predict the position of the focus accurately, it was necessary to experimentally characterise the position of the elements. This reduced the errors in position from over 3 mm to less than 1.5 mm. Although there was an effect on the phase of the source pressure, the crosstalk was not sufficient to disrupt the intended foci. However, the effect of this on aberration correction may be more significant and will be explored in future work. For this transducer, using an optimised element diameter rather than the nominal diameter had minimal effect, but this is unlikely to be the case for all transducers. The magnitude of these effects is dependent on the construction and behaviour of the transducer and driving system, and their characterisation is important for array transducers if a variety of steered fields and aberration corrections are to be simulated and applied in practice.

## Figures and Tables

**Table 1:** Table of errors averaged over ten steered fields. *ϵ p_f_* is the error in the focal pressure amplitude, *ϵ* position is the distance between the simulated and measured focus (position of spatial peak pressure), and the *l*_2_ relative error norm.

Size	Positions	*p*0 variation	Crosstalk	*ϵ p_f_*(%)	*ϵ* position (mm)	*l*_2_
✘	✘	✘	✘	20.9	3.3	0.75
✘	✘	**✓**	✘	20.8	3.3	0.75
✘	✘	**✓**	**✓**	3.5	3.1	0.51
**✓**	✘	✘	✘	19.7	3.3	0.74
**✓**	✘	**✓**	✘	19.6	3.3	0.74
**✓**	✘	**✓**	**✓**	4.2	3.1	0.51
✘	**✓**	✘	✘	21.6	1.1	0.57
✘	**✓**	**✓**	✘	21.5	1.2	0.59
✘	**✓**	**✓**	**✓**	3.3	1.4	0.35
**✓**	**✓**	✘	✘	20.4	1.0	0.55
**✓**	**✓**	**✓**	✘	20.3	1.2	0.58
**✓**	**✓**	**✓**	**✓**	4.0	1.5	0.37
